# The Value of the Electrocardiogram in Adult Congenital Heart Disease

**DOI:** 10.3390/jpm14040367

**Published:** 2024-03-29

**Authors:** William A. Schiavone, David S. Majdalany

**Affiliations:** 1 Cleveland Clinic Heart and Vascular Institute, Cleveland, OH 44120, USA; schiavw@gmail.com; 2 Department of Cardiovascular Diseases, Mayo Clinic, Phoenix, AZ 85054, USA

**Keywords:** electrocardiographic or electrocardiogram (EKG), adult congenital heart disease (ACHD)

## Abstract

The electrocardiogram is the first test that is undertaken when evaluating a patient’s heart. Diagnosing congenital heart disease in an adult (ACHD) can be facilitated by knowing the classical electrocardiographic (EKG) findings. These EKG findings often result from the congenital defect that prevents a part of the cardiac conduction system from occupying its normal anatomic position. When these classical EKG findings are not present, the clinician should consider alternate diagnoses. As the patient with congenital heart disease ages, with native anatomy or after surgical or device repair, the EKG can be used to assess the patient’s status and to decide if and when treatment requires adjustment. This is because the electrocardiogram (EKG) can diagnose the hypertrophy or enlargement in a cardiac chamber that results from the congenital defect or anomaly and can diagnose an arrhythmia that might compromise an otherwise stable anatomy. While ACHD often involves intracardiac shunting, in many cases the abnormality only involves cardiac electrical conduction block or ventricular repolarization. These life-threatening diseases can be diagnosed with an EKG. This review will demonstrate and explain how the EKG can be used to diagnose and follow adults with congenital heart disease. When coupled with history and physical examination, the value of the EKG in ACHD will be apparent. A diagnosis can then be made or a differential diagnosis proposed, before an imaging study is ordered.

## 1. Introduction

This review paper will list several types of ACHD and will characterize its anatomy and physiology for each of them. Patient symptoms will be discussed along with cardiac auscultation findings when helpful. A pertinent EKG tracing will be presented and then explained using a structured method in order to assess the rhythm, the atria, AV conduction, QRS morphology and contour, and repolarization changes.

## 2. Case-Based Approach

### 2.1. Atrial Septal Defect (ASD)

#### Anatomy and Physiology

**Figure 1:**
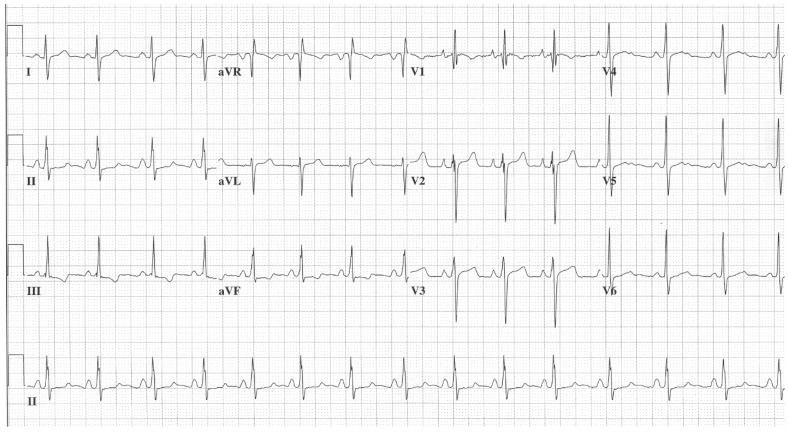
**EKG in ostium secundum atrial septal defect:** This is a 12-lead EKG with 1 rhythm strip undertaken on a 19-year-old male with an ostium secundum ASD. The EKG was undertaken with standard voltage (10 mm/mV) and paper speed (25 mm/s). The rhythm is sinus at 85/min and there is right atrial enlargement from the left to right shunt at atrial septal level. The QRS axis is mildly rightward and the QRS in V1 measures 95 msec and demonstrates an rsR’ pattern, fulfilling criteria for IRBBB. Additionally, there is a notched R wave (crochetage) in inferior leads II and aVF. The inferior ST segment abnormalities are related to right atrial enlargement. These EKG findings support the diagnosis of an ASD in the region of the fossa ovalis with RV volume overload, but not yet a significant RV pressure overload.

**Figure 2:**
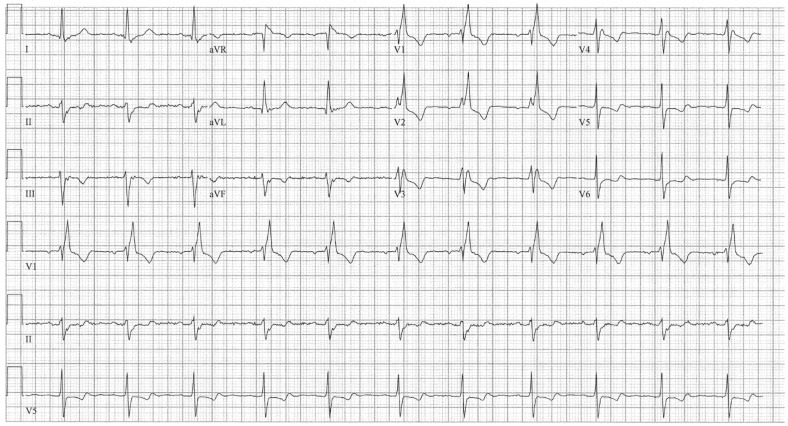
**EKG in ostium secundum atrial septal defect:** This is a 12-lead EKG with 3 rhythm strips undertaken on a 60-year-old female with an ostium primum ASD. The EKG was undertaken with standard voltage (10 mm/mV) and paper speed (25 mm/s). The rhythm is sinus at 70/min with a normal PR interval. There is an abnormal left QRS axis, as expected. There is a complete right bundle branch block, which can be seen in older patients with Ostium primum ASD. There are lateral precordial repolarization changes.

ASD has three common types which, together, are the most frequent heart defects observed in adulthood. These types are outlined as follows:

- Ostium secundum ASD is a defect in the region of the fossa ovalis. It is the most common type of ASD. Because the sinus node is in the normal position near the connection of the superior vena cava to the right atrium, it is the pacemaker of the heart and shows a sinus P wave. Additionally, because there is a normal position of the AV bundle (the bundle of His) the QRS axis is normal. An incomplete right bundle branch block (IRBBB) is 10 times more common among patients with an ASD than among healthy volunteers without cardiac disease, though there was no correlation between IRBBB and ASD diameter [1]. A notch (crochetage) in the R wave of the inferior leads (II, III and aVF) is noted seven times more often in patients with ASD than in healthy volunteers [1]. A significant correlation has been observed between ASD diameter and the presence of inferior R wave crochetage [1]. As a result of the ASD there is a left-to-right shunt that initially enlarges the right atrium and the right ventricle. This right ventricular volume overload, rather than real transmission delay, accounts for the rsR’ or rSr’ seen in patients with ASD. When the defect and the shunt are large, pulmonary hypertension can result. An ASD can permit a venous thromboembolism to become a paradoxical embolism. Cardiac auscultation of a patient with ostium secundum ASD is characterized by a systolic flow murmur in the pulmonary outflow tract and a wide-split and fixed-split S2. A wide- and fixed-split S2 means that A2–P2 (the components of S2) splitting is wide and does not vary with inspiration or with expiration. Figure 1 demonstrates a sample EKG from a patient with an ostium secundum ASD.

b. Ostium primum ASD is part of the AVSD spectrum discussed below. It is the second most common type of ASD. It is a defect low in the atrial septum; however, while there is no interventricular communication, there is a cleft leaflet of the left AV valve. An ostium primum ASD occasionally has P wave changes that are consistent with left or right atrial enlargement but will often have a first-degree AV block due to prolonged intra-atrial conduction. Because of the location of the septal defect, all patients with ostium primum ASD will have an abnormal left axis. In addition, an older patient can demonstrate complete right bundle branch block [2]. Like ostium secundum ASD, cardiac auscultation of an ostium primum ASD is characterized by a systolic flow murmur in the pulmonary outflow tract and wide- and fixed-split S2. The systolic murmur of AV valve regurgitation may or may not be audible. Figure 2 demonstrates a sample EKG from a patient with an ostium primum ASD.

c. Sinus venosus ASD is located in the upper atrial septum, where it connects to the superior vena cava. Of the three common types of ASD, it is the least common. It is often coupled with an anomalous right upper pulmonary vein that connects to the upper right atrium, rather than normally to the left atrium. Because of the location of the sinus venosus defect, the sinus node is absent or ectopic and the EKG rhythm is junctional or low atrial. Cardiac auscultation of a sinus venosus ASD has the same pulmonary outflow tract findings as ostium secundum and ostium primum type ASDs. These three types of ASD can be differentiated electrocardiographically.

### 2.2. Atrioventricular Septal Defect (AVSD)

#### Anatomy and Physiology

AVSD is also known as atrioventricular canal or endocardial cushion defect. AVSD is subdivided into partial AVSD and complete AVSD.

Partial AVSD includes an ostium primum atrial septal defect and a cleft anterior leaflet of the left AV valve. Partial AVSD is characterized by a holosystolic murmur of systemic AV valve regurgitation due to the cleft leaflet.

**Figure 3:**
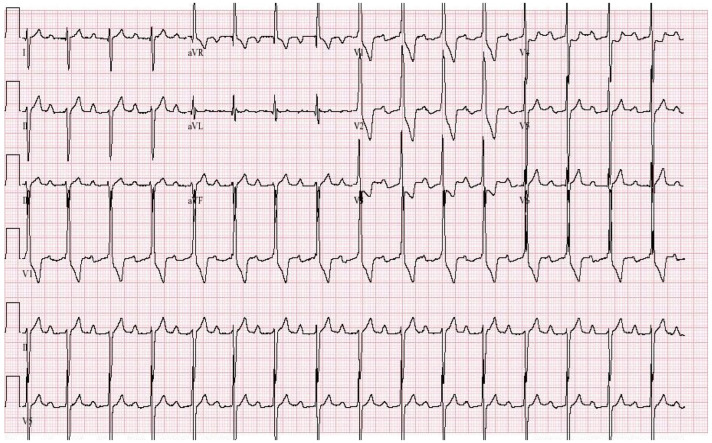
**EKG in complete atrioventricular septal defect:** This is a 12-lead EKG with 3 rhythm strips undertaken on a 19-year-old male with an uncorrected complete AVSD. The EKG was undertaken with standard voltage (10 mm/mV) and paper speed (25 mm/s). The rhythm is sinus at 90/min. The peaked P waves in II, III, aVF, V1 and V2 indicate right atrial enlargement. There is a first-degree AV block measuring 280 msec. In addition to the abnormal left axis from the ostium primum defect there is right ventricular hypertrophy (RVH) with secondary repolarization abnormalities. The RVH is due to volume and pressure overload and accounts for the large S wave in lead I, the tall R wave in aVR, the tall R waves in the right precordial leads and the deep S waves in the left precordial leads.

Complete AVSD includes a common AV junction coexisting with a deficient AV septation. The valve is shared and can have up to five leaflets, resulting in significant interatrial and interventricular communication. There is overload of the right atrium and the right ventricle and resultant congestive heart failure. A pansystolic murmur is auscultated due to atrioventricular valve regurgitation. Largely due to intra-atrial conduction delay, the PR interval is prolonged and there is first-degree AV block in most patients with AVSD [2]. Figure 3 demonstrates a sample EKG from a patient with a complete AVSD.

### 2.3. Left-Sided Valvular Heart Disease of Congenital Etiology

#### Anatomy and Physiology

Left-sided valvular heart disease of congenital etiology will be divided into three types, as follows:

Bicuspid aortic valve with severe aortic stenosis, bicuspid aortic valve with severe aortic regurgitation and myxomatous mitral valve disease with severe mitral regurgitation.

**Figure 4:**
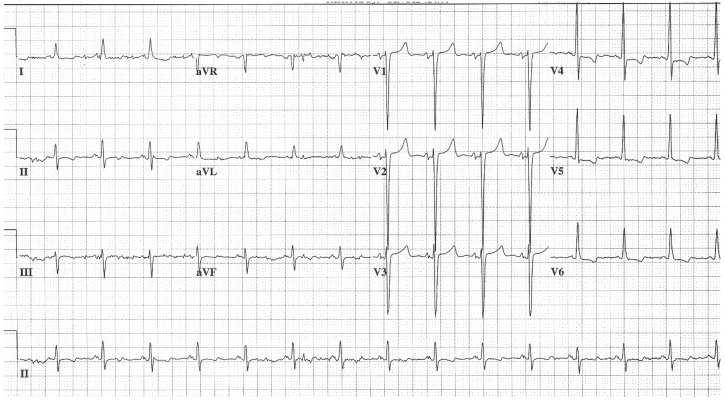
**EKG in bicuspid aortic valve with severe aortic stenosis:** This is a 12-lead EKG with 1 rhythm strip undertaken on a 50-year-old female with a bicuspid aortic valve with severe AS. The EKG was undertaken with standard voltage (10 mm/mV) and paper speed (25 mm/s). The rhythm is sinus at 85/min. The P waves are normal, as is the PR interval. There is LVH with secondary repolarization changes due to hypertrophy. This is the EKG of a left ventricular systolic pressure overload.

**Figure 5:**
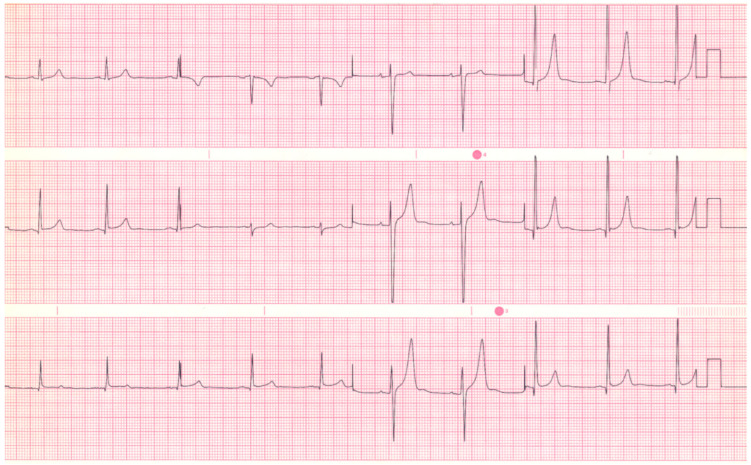
**EKG in bicuspid aortic valve with severe aortic regurgitation**: This is a 12-lead EKG undertaken on a 20-yar-old male with a bicuspid aortic valve with severe AR. The EKG was undertaken with standard voltage (10 mm/mV) and paper speed (25 mm/s). The rhythm is sinus at a rate of 58/min. The P waves and PR interval are normal. In the lateral precordial leads there is large R wave voltage with Q waves, mild ST segment elevation and tall asymmetric and peaked T waves. This is the EKG of a left ventricular diastolic volume overload. These findings are more reliably diagnostic for young patients than for those with advanced acquired disease and severe dilatation.

**Figure 6:**
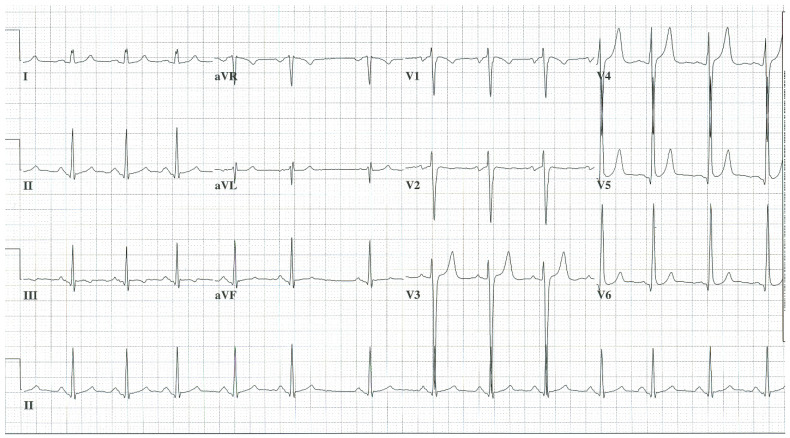
**EKG in myxomatous mitral valve disease with severe mitral regurgitation:** This is a 12-lead EKG with 1 rhythm strip undertaken on a 25-year-old male with MMVD with severe MR. The EKG was undertaken with standard voltage (10 mm/mV) and paper speed (25 mm/s). The rhythm is sinus with sinus arrhythmia. The P waves demonstrate left atrial enlargement and the PR interval is normal. There is LVH by voltage criteria and there are tall asymmetric and peaked precordial T waves. This is the EKG of a left atrial enlargement and a left ventricular diastolic volume overload.

- Bicuspid aortic valve with aortic stenosis (AS) is due to the restricted motion of this valve’s two (rather than the normal three) leaflets. During systole the aortic leaflets impede the left ventricular outflow because they cannot flatten against the ascending aorta. When the leaflet motion restriction is severe the pressure gradient across the aortic valve becomes large and the left ventricle must hypertrophy to overcome this large gradient. Auscultation of the heart of this patient demonstrates a crescendo/decrescendo systolic ejection murmur at the base of the heart and sometimes an early systolic ejection click. The EKG of a bicuspid aortic valve with severe AS shows left ventricular hypertrophy (LVH) with ST and/or T wave abnormalities secondary to hypertrophy, including ST segment depression and T wave inversion in leads I, aVL and V4–V6 (see Figure 4). When undiagnosed or untreated, the pressure-overloaded left ventricle can become dilated and dysfunctional. It is best to make the diagnosis before this left ventricular failure.

b. Bicuspid aortic valve with aortic regurgitation (AR) can be due to malcoaptation of the two leaflets of this congenital aortic valve deformity, with or without associated aortic root or ascending aortic dilatation. When chronic and severe, AR renders the left ventricle volume dilated and, if left untreated, results in systolic left ventricular dysfunction. Auscultation of the heart of a patient with bicuspid aortic valve with severe AR reveals an early systolic ejection click at the lower left sternal border and a decrescendo diastolic murmur at the base of the heart, often with presystolic accentuation at the cardiac apex (Austin Flint murmur). The EKG of a bicuspid aortic valve with severe chronic AR (see Figure 5) shows LVH with tall, upright T waves in the lateral precordial leads due to left ventricular volume overload. A bicuspid aortic valve, be it with predominant AS or AR, is present in 1–2% of the population [3], but certainly a minority of patients with bicuspid aortic valve will have severe valvular stenosis or regurgitation.

c. Myxomatous mitral valve disease (MMVD) or mitral valve prolapse is histologically characterized by myxomatous valve degeneration. MMVD or mitral valve prolapse is present in 2–3% of the population [4]. Most cases of MMVD are mild and asymptomatic. Myxomatous valve degeneration leads to valve redundancy which causes the leaflet(s) to prolapse, resulting in a mid-systolic click that can be followed by a mid-to-late systolic murmur at the cardiac apex. Myxomatous degeneration can be responsible for chordae tendineae rupture, resulting in a holosystolic murmur of mitral regurgitation (MR). Severe MR, when chronic, is responsible for left ventricular volume overload and, when untreated, can lead to left ventricular systolic dysfunction. The EKG of MMVD with severe chronic MR has left atrial enlargement and LVH by QRS voltage and with tall, upright T waves in the lateral precordial leads. Figure 6 is an ECG of a patient with MMVD with severe MR. Atrial fibrillation is a common presentation.

### 2.4. Congenital Complete Heart Block (CCHB)

#### Anatomy and Physiology

**Figure 7:**
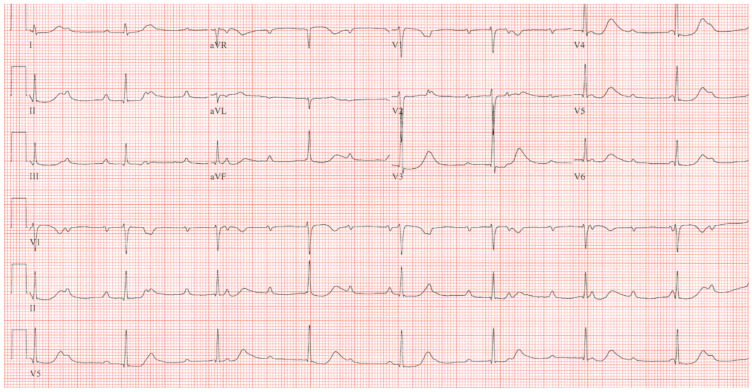
**EKG in Congenital Complete Heart Block:** This is a 12-lead EKG with 3 rhythm strips undertaken on an otherwise healthy 38-year-old female who presented to her physician with recurrent dizziness when standing still after running. She was understood to have had a slow heart rate for her entire life. The EKG was undertaken with standard voltage (10 mm/mV) and paper speed (25 mm/s). The rhythm is sinus at 100/min with junctional escape rhythm at 42/min. There is a 3rd-degree AV block. The QRS complexes have normal axis, duration and contour despite being independent from the P waves. There are no abnormalities of repolarization, including ST segments, T waves and QTc. Considering her lifelong bradycardia, all five diagnostic criteria for CCHB were met.

Congenital complete heart block (CCHB) is suspected in a young patient when there is a slow heart rate, often in the presence of no other congenital cardiac anomaly. Criteria used to make the diagnosis include atria and ventricles that beat independently of each other; a ventricular rate that is slower than the atrial rate; the lack of other pertaining cardiac rhythms; the lack of signs, symptoms, or history of generalized disease; and a patient that is not over 20 years of age. Permanent pacemakers were inserted in patients who presented with syncope. In a study that followed 14 patients for an average of 25 years, Pordon and Moodie [5] demonstrated the need for more widespread use of pacemaker implantation once patients reached adulthood. Michaelsson et al. [6] noted that ventricular rate in patients with CCHB decreased with age at a mean rate at 15 years of 46/min, at 16 to 20 years of 43/min, at 21 to 30 years of 41/min, at 31 to 40 years of 40/min, and after 40 years of age of 39/min. When exercising, a patient with CCHB will have an increase in ventricular rate in response to sympathetic discharge and increase in blood pressure due to muscle contraction. Figure 7 showcases the EKG of a patient with CCHB. At rest after exercise, as the ventricular rate decreases and the blood pressure loses support from muscle contraction, syncope can result if the patient does not sit or lie down.

### 2.5. Tetralogy of Fallot (ToF)

#### Anatomy and Physiology

**Figure 8:**
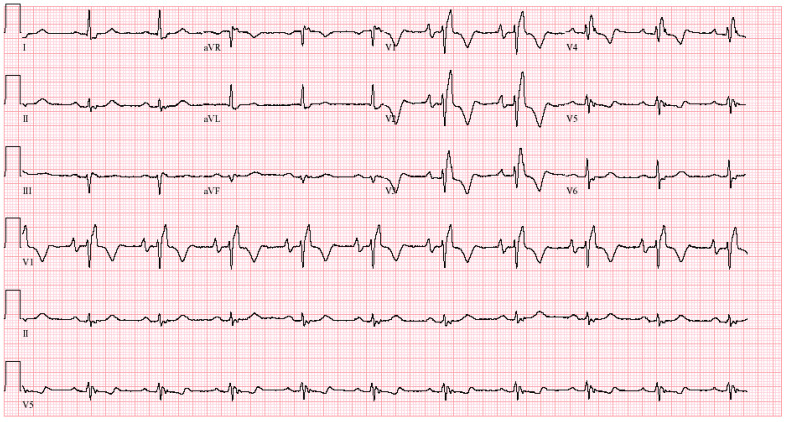
**EKG in tetralogy of Fallot post-repair:** This is a 12-lead EKG with 3 rhythm strips undertaken on a 46-year-old male with ToF that had been repaired 25 years prior to their presentation with palpitations. The EKG was undertaken with standard voltage (10 mm/mV) and paper speed (25 mm/s). The rhythm is sinus at 63/min with right atrial enlargement best seen in the right precordial leads. The PR interval is mildly prolonged at 220 ms. There is right bundle branch block with a QRS duration of 180 ms. Holter monitoring demonstrated that his palpitations were due to non-sustained ventricular tachycardia. The symptoms, ventricular tachycardia, QRS prolongation and his prominent murmur of pulmonary regurgitation led to pulmonary valve insertion.

Tetralogy of Fallot (ToF) is a cyanotic congenital heart disease that represents almost 10% of congenital heart diseases. It consists of anterocephalad deviation of the outlet septum, narrowing of the pulmonary tract, large outflow tract ventricular septal defect, aorta overriding the interventricular septum, and concentric hypertrophy of the right ventricle [7]. Surgical repair has become routine practice in pediatric heart surgical centers and, as a result, there is a sizeable population of adults with repaired ToF. Although surgical repair enlarges the pulmonary outflow tract, ameliorates pulmonary valve stenosis, and patches the ventricular septal defect, residual pulmonary regurgitation and/or stenosis can worsen. Although these residual lesions are tolerated for a long time, right ventricular remodeling and associated arrhythmias, exercise limitation, heart failure and even death can result. The EKG of adult patients with ToF after surgical repair reflect this progressive right ventricular dilatation and dysfunction [2]. With increasing age, the right atrium increases in size and atrial arrhythmias follow. The PR interval can increase and, due to the ventricular septal defect closure, complete atrioventricular block may occur. The QRS almost always demonstrates right bundle branch block. The main risk factor for ventricular arrhythmias in adults with repaired ToF is QRS prolongation, particularly when the QRS duration is ≥180 ms [8]. Non-sustained ventricular tachycardia can result and must be sought when patients have such QRS prolongation or symptoms of palpitations or syncope. Pulmonary valve replacement to correct the pulmonary regurgitation can be beneficial. Additional risk factors of ventricular arrhythmias in ToF include QRS dispersion, an increase in QRS duration per year, QT duration and QT dispersion. Electrophysiologic studies have identified macro-reentry as the major mechanism for induced and spontaneous anatomic isthmuses related to VT. Moreover, these anatomic isthmuses are bordered by patch material, valve annuli, and surgical incisions [9]. Figure 8 demonstrates the EKG of a patient with repaired tOF.

### 2.6. Congenitally Corrected Transposition of the Great Arteries (CCTGA)

#### Anatomy and Physiology

Congenitally corrected transposition of the great arteries (CCTGA), also known at L-TGA, is a rare congenital heart defect. It comprises less than 1% of congenital heart disease [2]. It is characterized by atrioventricular and ventriculoarterial discordance. These two anomalies “correct” each other. As a result, although the ventricles are switched, the blood is properly oxygenated and distributed. The cyanosis that would occur in the presence of only one of the discordances is corrected by there being two discordances. The patient with CCTGA will often remain asymptomatic and not diagnosed until adulthood, or even later in life. What often brings patients with CCTGA to recognition is the inability of the morphological right ventricle to support the systemic circulation. This overloaded systemic ventricle becomes dilated and dysfunctional, resulting in systemic atrioventricular valvular (as the AV valve remains with its anatomic ventricle, this is the tricuspid valve) regurgitation and heart failure. Physical examination demonstrates a prominent holosystolic murmur at the apex.

**Figure 9:**
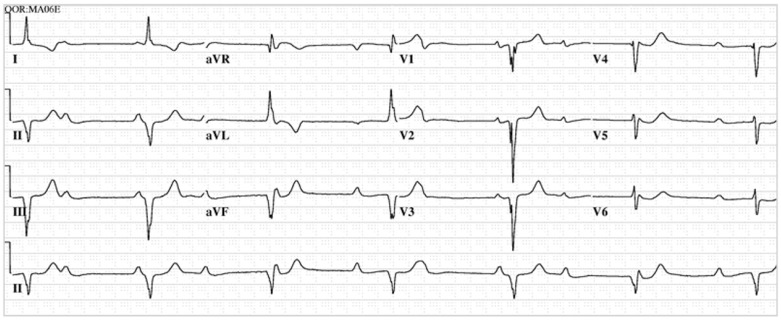
**EKG in Congenitally Corrected Transposition of the Great Arteries:** This is the 12-lead EKG with one rhythm strip of a 26 year-old male who complained of episodic dizziness. The EKG was undertaken with standard voltage (10 mm/mV) and paper speed (25 mm/s). The rhythm is sinus at 68/min. The ventricular rate is ~38/min. There is 3rd degree AV block. There is an abnormal left QRS axis (frankly there are Q waves in leads II, III and aVF). QRS duration is 100 ms. Characteristic of CCTGA, the right precordial QRS complexes have Q waves and the left precordial QRS complexes have no Q waves. A permanent pacemaker was required.

Because of the malposition of the AV node and bundle of His in CCTGA, AV conduction is prone to complete AV block at an incidence of 2–3% of patients per year (Figure 9). The QRS complexes in CCTGA are characterized by Q waves in the right precordial leads and absent Q waves in left precordial leads. This is because the ventricles and associated bundles are reversed, causing septal activation to occur from right to left [2].

### 2.7. Hypertrophic Obstructive Cardiomyopathy (HOCM)

#### Anatomy and Physiology

Hypertrophic obstructive cardiomyopathy (HOCM), also known as idiopathic hypertrophic subaortic stenosis (IHSS), is an inherited myocardial disease. The broader category of hypertrophic cardiomyopathy has an incidence of approximately 1 in 500 people.

**Figure 10:**
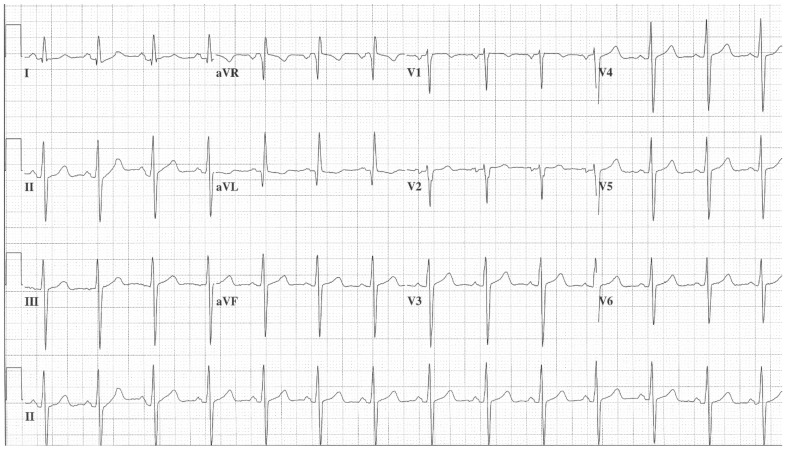
**EKG in Hypertrophic Obstructive Cardiomyopathy (HOCM):** This is the 12-lead EKG with one rhythm strip of a 29-year-old woman who presented with recurrent exertional syncope. As a result, she was not able to corral her toddler without fear of falling. The EKG was undertaken with standard voltage (10 mm/mV) and paper speed (25 mm/s). Her systolic murmur was dynamic. It increased with walking, with Valsalva strain, and when standing from squatting. The rhythm is sinus at 85/min with normal P wave morphology and PR interval. There is an abnormal left QRS axis. The QRS duration is normal at 80 ms. The sum of R wave in aVL + S wave in V3 = 30 mm, which meets the Cornell criteria for LVH in a woman. Characteristic of HOCM, the Q wave in lead aVL suggests greater upper anterior septal thickness. The patient received effective treatment.

HOCM is a type of hypertrophic cardiomyopathy (HCM) that is characterized by proximal thickening of the ventricular septum of at least 15 mm that is not a result of abnormal loading conditions. This septal thickening narrows the left ventricular outflow tract and results in a gradient of at least 30 mmHg. Usual symptoms include dyspnea, chest pain, palpitations and post-exertional syncope [10]. Physical examination is heralded by a systolic murmur that increases with Valsalva strain, decreases with squatting and roars when standing from squatting. The diagnosis is confirmed by imaging. The EKG in HOCM may have abnormal Q waves, simulating myocardial infarction with deeper than normal Q waves in lateral and inferior leads [11]. Although Q waves can be present in both obstructive and nonobstructive forms of HCM, a cardiac magnetic resonance study revealed that abnormal Q waves were associated with greater upper anterior septal thickness [12]. A convolutional neural network trained and validated using a digital 12-lead EKG from patients with a verified HCM diagnosis, and non-HCM age- and sex-matched control subjects, was able to differentiate HCM from normal with high diagnostic performance [13]. EKG voltage criteria for left ventricular hypertrophy are nearly always present in patients with HCM [11] (Figure 10).

The left atrium in patients with HOCM enlarges over time and predisposes to atrial fibrillation and heart failure. Some patients are at risk for sudden cardiac death. There are effective treatments for patients diagnosed with HOCM.

### 2.8. Apical Hypertrophic Cardiomyopathy (Apical HCM)

#### Anatomy and Physiology

**Figure 11:**
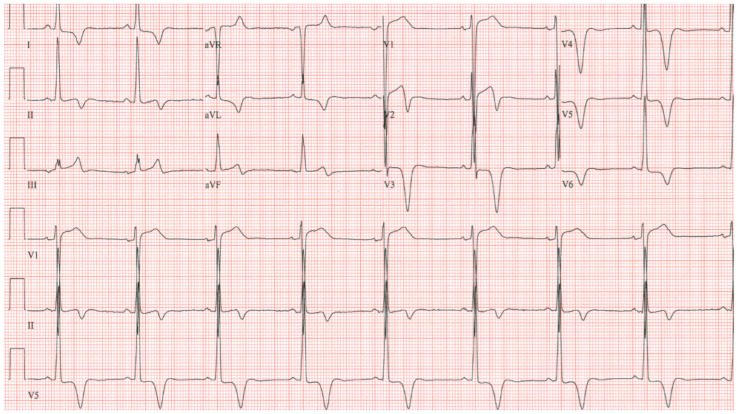
**EKG in apical hypertrophic cardiomyopathy:** This is the 12-lead EKG with 3 rhythm strips of a 24-year-old male applying for a job as a policeman. He was asymptomatic and had no murmur or gallop. The EKG was undertaken with standard voltage (10 mm/mV) and paper speed (25 mm/s). By voltage criteria for LVH, the sum of S wave in V1 and R wave in V5 is >50 mm. The negative T wave in V3 measures nearly 15 mm. These meet the criteria for apical HCM. The QRS duration is 80 ms. The rhythm is sinus at ~50/min with normal P wave morphology and a normal PR interval. These corroborate his lack of dyspnea. There is no family history of sudden cardiac death. It will be difficult to decide if this is the right work for this man in the long term.

Apical hypertrophic cardiomyopathy was described in 1979 by Yamaguchi et al. [14] and this type of HCM bears the author’s name (Yamaguchi syndrome). It is characterized by concentric apical hypertrophy that has a spade-like configuration when observed in the right anterior oblique ventriculogram at end-diastole and in the long axis two-dimensional echocardiogram. This marked hypertrophy results in a relatively small-volume left ventricular chamber that requires elevated left ventricular end-diastolic pressure to accommodate the volume. Left atrial enlargement and atrial fibrillation often result. Patients with apical HCM may have dyspnea on exertion and chest pain in the absence of significant coronary artery disease. Apical HCM does not demonstrate left ventricular outflow tract obstruction like is seen in HOCM. The EKG shows giant symmetric negative T waves (>10 mm) associated with high QRS voltage (R wave > 26 mm in lead V5 or the sum of the S wave in V1 and the R wave in V5 ≥ 35 mm) [14,15] (Figure 11). The depth of the T wave may be associated with maximal apical thickness measured by magnetic resonance imaging [12]. In a single-center study of 160 HCM patients with an LV apical aneurysm [16], diagnosed by echocardiography, cardiac magnetic resonance, or both, 63% of the study cohort had apical HCM. Increasing LV apical aneurysm size confers a poorer prognosis.

### 2.9. Ebstein Anomaly

#### Anatomy and Physiology

Ebstein anomaly (EA) is characterized by an apically displaced tricuspid valve that is also deformed. Ebstein first described this congenital heart disease at an autopsy in 1866 [2]. EA accounts for <1% of all congenital heart disease. As a result of the apically displaced tricuspid valve, the right ventricle becomes atrialized and there is often severe tricuspid regurgitation. In 80% of patients with EA an ASD or patent foramen ovale is present. The combination of severe tricuspid regurgitation and decrease in right ventricular volume leads to a compromise of right ventricular function and low cardiac output. Surgical tricuspid valvuloplasty is a difficult operation but can be beneficial in EA, with the cone procedure being the most recent technique. Patients with EA usually have sinus rhythm, but atrial arrhythmias are common due to the large right atrial size. Supraventricular tachyarrhythmias (SVT) are the most common presentation in adults with EA. In an EKG study of 26 patients with EA, 3 presented with junctional tachycardia, 4 presented with atrial flutter and 2 presented with atrial fibrillation [17].

**Figure 12:**
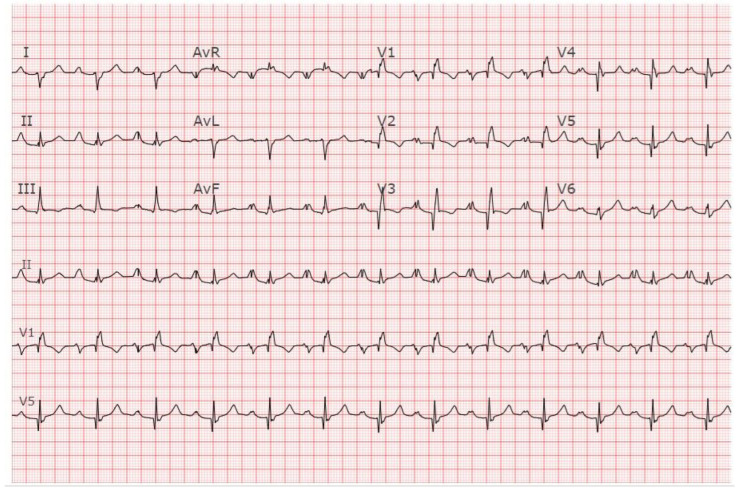
**EKG in Ebstein anomaly:** This is a 12-lead EKG with 3 rhythm strips of an 18-year-old female who presented with reduced exercise tolerance. The tracing was recorded at 25 mm/sec and standard voltage (10 mm/mV). The rhythm is sinus at 75/min. There is right atrial enlargement. Notice the change in P wave morphology from a single upright P wave to a bifid P wave when recorded in the Lead II rhythm strip. There is 1st-degree AV block. The PR interval measures 290 ms and remains constant. There is an abnormal right axis. There is incomplete right bundle branch block with Q waves in all of the precordial leads but for V6. The precordial R waves have low amplitude.

The EKG in EA shows tall P waves that reflect the large right atrium. Because of intra-atrial conduction delay, there is often first-degree AV block. The QRS in EA shows incomplete or complete right bundle branch block with low-amplitude R waves in the right precordial leads due to the small-volume right ventricle (Figure 12). In ~20% of patients with EA, an accessory pathway, usually around the abnormal tricuspid annulus, is present. This can result in a short PR interval with delta waves. SVT with an accessory pathway can lead to rapid conduction of atrial fibrillation or atrial flutter to the ventricles, which might cause sudden cardiac death. For this reason, catheter ablation should be performed in patients with EA when SVT coincides with one or multiple accessory pathways.

### 2.10. Pulmonary Valve Stenosis (PS)

#### Anatomy and Physiology

Pulmonary valve stenosis is a congenital valvular heart disease. PS is a component of other congenital heart diseases, such as ToF, but in this section PS as a solitary abnormality will be discussed. Valvular PS is characterized by the fusion of pulmonary valve leaflets and doming of the pulmonary valve during systole, narrowing the egress of blood from the right ventricle and requiring the right ventricle to eject its stroke volume across a valve with a large gradient. This results in right ventricular hypertrophy to meet the increased workload. Symptoms can include fatigue, dyspnea on exertion, exertional syncope and chest pain. The physical examination features a crescendo–decrescendo systolic murmur and a left parasternal heave.

**Figure 13:**
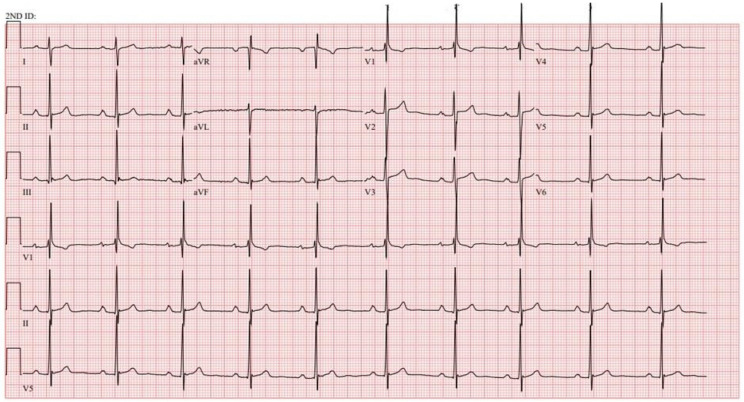
**EKG in severe pulmonary valve stenosis**: This is the 12-lead EKG with 3 rhythm strips of a 33-year-old male who presented with exertional dyspnea and was noted to have severe valvular PS. It is recorded with standard voltage (10 mm/mV) and 25 mm/sec paper speed. The heart rate is ~60/min. The P waves are peaked in leads II, III and aVF suggesting right atrial enlargement. The PR interval is at the upper limits of normal at 200 ms. There is an abnormal right axis with dominant S waves in leads I and aVL. The V1 pattern is rsR’ and there is incomplete RBBB and no ST and T wave abnormalities, the most common EKG presentation found in PS by Fazelifar [18].

The EKG changes of severe right ventricular hypertrophy include tall R waves in right precordial leads, deep S waves in left precordial leads, right axis deviation, increased P wave amplitude due to right atrial enlargement (see Figure 13) and right ventricular strain pattern. Fazelifar et al. [18] studied the EKGs of 49 patients with severe pulmonary stenosis (PS) (gradient > 60 mmHg) whose average age was 29 years. The rhythm was sinus in all 49 patients. The heart rate averaged 76/min. The PR interval was normal in all of the patients. The most common V1 pattern was rsR’ in 34%, followed by monophasic R in 26%, and rS in 20%. The QRS duration ranged from 100–132 ms. Incomplete RBBB was present in 33%, complete RBBB in 29%, intraventricular conduction delay in 10%, and normal in 28%. The limb lead with the largest voltage S wave was lead I. It was concluded that the effects of severe PS on the EKG are in the QRS (depolarization changes). This was contrasted with a group of patients with pulmonary hypertension in which the predominant effects on the EKG were ST segment depression and T wave inversion (repolarization changes).

## 3. Conclusions

Skillful diagnosis of the EKG can uncover a hidden diagnosis of ACHD and be used as a tool to follow a patient in whom the diagnosis has been made. Making a diagnosis can lead to improvement in the patient’s clinical course.

The use of artificial intelligence (AI) to screen an EKG for certain diagnoses is upon us. We have learned that AI can be used to detect HCM [13]. Elias et al. [19] have also shown that an EKG deep learning algorithm can be used to detect left-sided valvular heart disease. Their algorithm for detecting left-sided valvular heart disease (with echocardiographic validation) was most accurate for AS, and sequentially less accurate for MR and for AR. The area under the receiver–operating curves were 0.88 for AS, 0.83 for MR, and 0.77 for AR.

The various anatomic anomalies of ACHD offer a challenge when interpreting the EKG as the cardiac lesions demonstrate heterogeneity and there could be overlap in EKG findings amongst different diagnoses. Changes in cardiac position such as mesocardia and dextrocardia can further alter EKG findings such as QRS axis. Here the clinician who has good EKG skills can recognize classical patterns and follow how symptoms and physical examination corroborate with the EKG, explaining anatomy and physiology. When assessing an EKG for left-sided valvular heart disease by deep-learning analysis, the differentiation among severe AS, severe AR and severe MR does not appear to be as robust as a structured EKG assessment by a clinician, especially when cardiac auscultation is included. In summary, when history and physical examination is coupled with knowledge of anatomy and physiology of CHD and sharp EKG interpretation skills, a diagnosis can be made, or a differential diagnosis can be proposed. Imaging testing can then be ordered with more efficiency.

## Data Availability

Not applicable.
